# Developmental Screening of Children with Congenital Hypothyroidism Using Ages and Stages Questionnaires Test

**Published:** 2019

**Authors:** Zahra RAZAVI, Setila DALILI, Mohammad Kazem SABZEHEI, Arman YOUSEFI, Shahla NOURI, Mahbubeh ABEDI, Hassan BAZMAMOUN

**Affiliations:** 1 Department of Pediatric Endocrinology, Hamadan University of Medical Sciences, Hamadan, Iran.; 2 Department of Pediatric Endocrinology, Pediatric Growth Disorders Research Center, 17 Shahrivar Hospital, School of Medicine, Guilan University of Medical Sciences, Guilan, Iran.; 3 Department of Neonatology, Besat Hospital, Hamadan University of Medical Sciences, Hamadan, Iran.; 4 Department of Pediatrics, Hamadan University of Medical Sciences.; 5 Department of population and family health, Hamedan University of Medical Sciences, Hamedan, Iran.; 6 Family Health Center, Hamadan University of Medical Sciences, Hamadan.; 7 Department of Pediatric Gastroenterology, Hamadan University of Medical Sciences, Hamadan, Iran.

**Keywords:** Congenital hypothyroidism, Levothyroxine, Permanent hypothyroidism, Transient hypothyroidism, ASQ test

## Abstract

**Objectives:**

Congenital hypothyroidism (CH) is one of the most common causes of mental retardation in children. We investigated the developmental status of children with CH screened by Ages & Stages Questionnaires (ASQ) measurement scores.

**Materials & Methods:**

In this retrospective study, neurodevelopmental status of 78 children diagnosed with CH followed up at the Outpatient Pediatric Endocrinology Clinic of Besat Hospital, Hamadan, Iran from May 2006 to Mar 2013, was evaluated by ASQ method. Data on age, sex, birth weight, birth length, head circumference, residency location, parental education level, primary venous TSH and T4 levels, age at diagnosis, treatment start age and initial levothyroxine dosage were extracted from medical records. Data were analyzed using statistical software SPSS. *P*-value less than 0.05 was considered statistically significant.

**Results:**

Of the 78 patients, 34 (43.6%) were female, 32 (41%) had developmental disorder, and 56 (71.8%) were living in urban areas. Types of developmental impairments included: global motor delay in 13 (40.6%) patients, problem-solving in 11 (34.3%), impaired communication skills in 5 (15.6%), impaired fine motor skills in 2 (6.2%), and impairment of personal social skills in 1 (3.1%). The average ages for diagnosis and treatment were 25.65 days in patients with developmental impairment and 17.99 days in those without developmental delay. ASQ results showed significant statistical correlation with initial dose of levothyroxine (*P*=0.017), age of hypothyroidism diagnosis (*P*=0.002) and age of treatment initiation (*P*=0.018).

**Conclusion:**

Early diagnosis and treatment along with initial levothyroxine dose were most important factors of ASQ scores of children with CH. Higher dose of the levothyroxine is required at onset.

## Introduction

“Congenital hypothyroidism has been introduced as one of the most common preventable causes of mental retardation which occurs in 1/3000 neonates” (1). Early identification and treatment within 2 wkof age can maintain normal cognitive development (2, 3). 

CH can be classified into permanent and transient types. Permanent CH can occur because of thyroid mal-development (ectopic, hypoplasia, and agenesis), an inborn error of thyroid metabolism, and central hypothyroidism. Whereas the transient type of the disease is attributable to the transplacental passage of maternal anti-thyroidal medication, maternal thyrotropin receptor blocking antibody (TRB-Ab), gene mutation, or iodine deficiency (4-7). 

Neurodevelopmental outcome is inversely related to the age of diagnosis and treatment of congenital hypothyroidism (2). The main objective treatment of hypothyroidism is to maintain normal growth and neurodevelopment of affected children. Therefore, close follow up and clinical evaluation along with frequent measurements of serum T4 and thyroid stimulating hormone (TSH) should be performed every few months during the first three years of life. 

The physical growth outcome can be evaluated by growth chart (8) and neurodevelopment can be assessed by a wide variety of tests in toddler and preschool-aged child.

 Ages & Stages Questionnaires (ASQ) includes developmental screening tests that can help parents check their child's development (9, 10). ASQ so far has been translated into many different languages and also recognized as a valid and strong tool for assessing and screening developmental status of children (11). This questionnaire has been translated into Persian in order to adapt it to the Iranian population. 

Screening programs of congenital hypothyroidism dramatically improved the neuropsychological outcome in affected children. However, mild impairments in neurocognitive function and intellectual sequel have been reported in some studies of early-treated CH children particularly in those with marked retardation of bone age and/or a low circulating thyroxin before treatment (12-15). 

We aimed to screen the developmental skills of 3 to 5 yr old children with congenital hypothyroidism detected by neonatal screening program in our region. 

## Material & Methods

Neonatal screening for congenital hypothyroidism was introduced in Iran in May 2005. This cross-sectional case study was conducted on children diagnosed with congenital hypothyroidism followed up in Pediatric Endocrinology Clinic of Besat Hospital, a tertiary care center in Hamadan, Iran, from May 2006 to Mar 2013. The study enrollment started in Mar 2013 and was carried out for two years. 

ELISA was method of screening to measure TSH on filter paper at day 3-7 of birth. Venous blood for total T4/ free T4 and TSH was obtained to confirm the diagnosis for those with abnormal result (TSH>5 mIU/L). Electrochemiluminescence was used to measure Total T4 and TSH.

 Criteria for patient recruitment were children diagnosed with CH (T4 < 6.5 µg/dL and thyroid-stimulating hormone (TSH)> 10 mIU/mL after one month of age) (7) followed up at the outpatient Pediatric Endocrinology Clinic of Besat Hospital, Hamadan, Iran

Data on age, sex, birth weight, birth length, head circumference, location of residence, parental education level, primary venous TSH and T4 levels, diagnosis age, age of initiation of treatment, initial levothyroxine (L-thyroxin or LT4) dosage, mean TSH level at first year and first three years after treatment start and number of annual visits were extracted from medical records. Congenital hypothyroidism cases were eligible for participation in this work if they had been treated with LT4 and were followed up closely in the ﬁrst three years of life. In accordance with guidelines of American Academy of Pediatrics, T4 < 6.5 µg/dL and TSH > 10 mIU/mL after one month of age was considered as CH (7). Children were diagnosed as permanent CH if they had serum TSH above 10 mIU/L during the first three years of treatment or if they needed LT4 therapy beyond 3 yr of age (TSH rise > 6 mIU/ml with temporary discontinuation of LT4 after the age of 3 yr (7, 16). 

Those cases lacking necessary data for the current work, and cases that did not have regular follow-up for the ﬁrst three years of life were excluded from the study. All children at least three years or more and with the criteria of the study were assessed by ASQ method. The patients were divided into three age groups; 3, 4, and 5 yr old. 

Ages & Stages Questionnaire is a developmental screening tool to determine whether a child requires further and more comprehensive evaluation/assessment designed for use by early educators and health care professionals. It has 20 questionnaires that correspond to age intervals from birth to 6 years. Each questionnaire contains simple questions for parents to answer about activities that their child is (or is not) able to do. The answers are assessed in five domains including communication, gross motor, fine motor, problem-solving and personal social skills (9, 10). The questionnaires are scored by converting each response to a numerical value: 

Z (most of the time) = 0

V (sometimes) = 5

X (rarely, never) = 10

If a parent identifies an item as a concern (circles to far right on form), an extra 5 points are scored for that item. Once the total score is calculated, if that score is higher than the cut-off score, the screening results suggest the child should be referred to child development specialist for a developmental checkup.

**Statistical analysis**

The Chi-square test was employed to analyze qualitative (categorical) variables expressed as ratio and percentages. For analyzing quantitative data, their normality of distribution was assessed based on mean and standard deviations. In order to compare qualitative variables between groups, chi-square test, and Fisher's exact test were used. To compare quantitative factors in two groups in the case of normality, independent t-test and for variables where the distributions of scores differed significantly from the normal distribution, Mann-Whitney U tests were used. All analyses were performed with statistical software SPSS ver.16 (Chicago, IL, USA). A *P*-value less than 0.05 was considered statistically significant. 

**Ethics approval**

The Ethics and Research Committee of Hamadan University of Medical Sciences approved the study, No: IR.UMSHA.REC.1394.380**. **Written informed consent was obtained from parents of children. 

## Results

**Figure 1:**
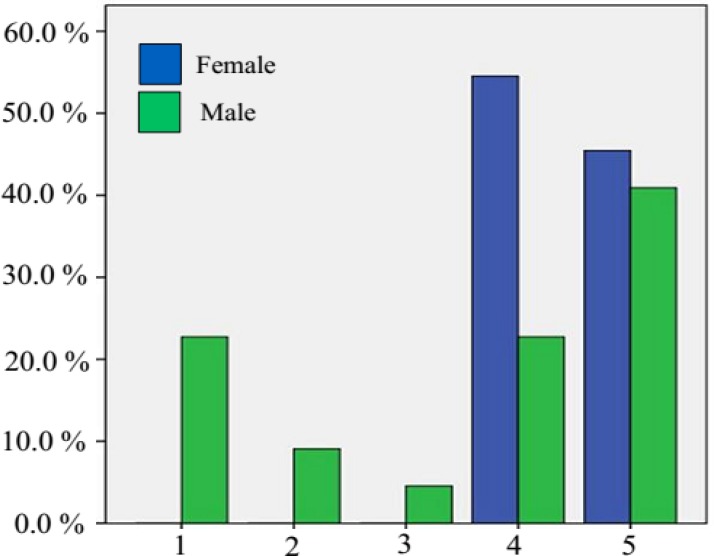
Frequency of impairment of five domains screened by ages & stages questionnaires or ASQ (1. communication, 2. fine motor, 3. personal-social, 4. problem-solving, 5. global)

Of 78 children, 34 (43.6%) were female, 56 (71.8%) were living in urban areas, and 22 (28.2%) were living in rural areas. Demographic characteristics of included subjects are summarized in Table 1. Five domains were evaluated using the ASQ, including communication, gross motor, fine motor, problem-solving, and personal-social skills. Of the 78 children, 3 to 5 yr old with congenital hypothyroidism, 32 patients (41%) had elevated ASQ score. The frequency of these five impairments is depicted in Figure 1.

Problem-solving skills was the most frequent type of developmental impairment among female children, while the global motor was the most prevalent one among male children. 

Age at diagnosis, initial dose of Levothyroxine (μgr/kg/d) and mean age at start of T4 supplementation age were the three variables that differed significantly between children with a normal ASQ test and those with an impaired ASQ test. Comparison of laboratory and treatment-related variables of subjects with normal ASQ score and those with elevated ASQ score are explained in Tables 2 and 3.

Those treated with initial dose LT4 ≤12 µgr/kg/d at diagnosis had a higher ASQ score (impaired) compared with those treated with a dose ≥ 12 μgr/kg/d (*P*=0.017) (Table 3). In those with normal ASQ, confirmation of the diagnosis was established at average 17.9 d and commencement of therapy with LT4 was at average 20.2 d. Whereas, in the group with impaired ASQ it was at average 25.65 and 26.59 d respectively. Laboratory and treatment-related variables are explained in Table 2.

No significant differences were found in gender, living location, birth length, weight, head circumference, parental consanguinity, parental education, primary venous TSH and T4 levels, and number of annual visits between two groups. Apart from initial dose of LT4, there was no significant association between the elevated ASQ score and mean of serum TSH, T4, and mean dose of levothyroxine (μgr/kg/d) for first year and 3 yr follow up. 

The association between the type CH (transient or permanent) and the development status was investigated using the Chi-square Test. There was not a significant correlation between type of CH and ASQ scores (*P*=0.74) (Table 4).

**Table 1:** Demographic characteristics of included subjects according to ASQ results (n=78)

Variable	State	Result of ASQ		P-value
		Impaired (n=32)	Normal (n=46)	
Sex	Female	11	23	0.1
	Male	21	23	
Place of living	Urban	20	36	0.2
	Rural	12	10	
Parental consanguinity	Yes	8	15	0.3
	No	24	31	
Maternal education	High school	10	10	0.5
	Diploma	16	23	
	Academic	6	13	
Paternal education	High school	4	5	0.6
	Diploma	18	21	
	Academic	10	20	
Mean maternal age (year)		26.5 (5.0)	27.4	0.37
Mean birth length (cm)		49.2	49.1	0.23
Mean birth weight (gr)		3223.9	3076.6	0.35
Mean birth head circumference (cm)		35.9	36.0	0.89

**Table 2:** Comparison of laboratory and treatment-related variables of participants with normal and impaired ASQ (n=78)

**Variable**	**Result of ASQ**		***P*** **-value**
	**Normal**	**Impaired**	
Diagnosis age (day)	17.95	25.65	0.002
Initial treatment age (day)	20.21	26.59	0.018
Initial blood TSH (mIU/ml)	38.86	40.42	0.76
Mean TSH for first year (mIU/ml)	4.6	5.31	0.26
Initial blood T4 (μg/dL)	5.60	4.99	0.39
Mean TSH for 3 yr (mIU/ml)	2.56	2.96	0.48

**Table 3:** ASQ results according to initial dose of LT4

Initial LT4 dose (μg/kg/d)	**ASQ result** ** (Number** ** and %)** **N=64**		**Df**	**OR**	**95%CI**		***P*** **-value**
	**Normal**	**Impaired**			U	L	
<12	19 (43.1)	22 (68.8)	1	3.1	8	1.2	0.017
≥12	27 (57.7)	10 (31.2)		2	0	0	

**Table 4:** Association between the types of congenital hypothyroidism (CH) and development status (n=78)

**The Type of CH**	**ASQ results**			***P*** **-value**
	**Impairment** **Number (%)**	**Normal** **Number (%)**	**Total** **Number (%)**	
Transient	5 (45.5)	6 (54.5)	11 (100)	0.74
Permanent	27 (40.3)	40 (59.7)	67 (100)	
Total	32 (41)	46 (59)	78 (100)	

## Discussion

The early detection and treatment along with adequate L-thyroxin dose lead to normal or near-normal neurocognitive outcomes in children with congenital hypothyroidism (17). Despite this, several studies have shown subnormal cognitive and motor development of timely treated congenital hypothyroidism (17-20).

In the present study, ASQ score was subnormal in 41% of early treated children with congenital hypothyroidism (elevated ASQ score). Problem-solving and global motor were the most frequent types of developmental impairment among female and male respectively. This is a surprising and unexpected finding. We assume the reason for high rate of abnormal ASQ is that ASQ is a developmental screening not diagnostic test that can help parents check their child's development and mental problem. In the next step, these children should be evaluated using standard IQ measurement scores. The results of further diagnostic tests may confirm or refute our findings. 

The associations between ASQ score and some demographic and laboratories’ variables were also assessed. We could not find any relation between the birth length, birth weight, birth head circumference, maternal age at pregnancy, sex, place of life, relation of parents, and parents’ education. Unlike our findings, parental education influenced intellectual development of the children with congenital hypothyroidism (21).

In this study, there was no relationship between biochemical severity (TSH and T4 levels at diagnosis) and ASQ scores. This finding is similar to the results of other studies (2, 22). No significant association was found between the severity of CH and intelligence coefficient (IQ) of patients with CH (23).

In contrast to our study, CH severity was correlated primarily with motor test results and reduced IQs (24, 25). A negative significant correlation was reported between initial TSH level and IQ scores among Dutch CH patients (26). On the other hand, no association was found between mild congenital hypothyroidism (TSH levels less than 15 mIU/L) and psychomotor development of preschool children in Belgium (27).

Similar to other studies (28-31), we could not find a significant difference between the two groups of impaired and normal ASQ scores in terms of the type of hypothyroidism (transient and permanent). This implies that adequate amounts of thyroid hormones early in life is essential for normal neurocognitive development. In contrast, transient thyroid dysfunction in the newborn was not associated with impaired psychomotor development (27).

Based on the evidence of this study, initiation of therapy with higher dose of LT4 (12 µgr/kg/d) was associated with a more favorable prognosis in ASQ score and intellectual development of congenitally hypothyroid children suggesting that initial L-thyroxin is an important factor for subsequent intellectual development of congenitally hypothyroid children.

In support of studies (2, 28), we believe that in order to achieve a good outcome, treatment of congenital hypothyroidism should be started with higher dose of LT4 than previously recommended. 

In this study, the mean age of diagnosis and treatment initiation was 20 days (these two variables were very close in their values because the treatment normally begins after the diagnosis) represents that diagnosis and start of treatment in CH infants were slightly later than recommended time frame of 14 days (3). Confirming previous studies (3, 16, 13, 17, 24) we assume that treating CH infants before 2 wk of age can give rise to better cognitive or motor outcome. Despite this, a study of Dutch children with congenital hypothyroidism found that advancing commencement of therapy from 28 days to 20 days after birth cannot fully normalize intellectual performance of affected children (32). 

Undoubtedly, our study has several limitations that need to be considered. First, we measured the cognitive outcome in terms of developmental status screening and not in terms of IQ confirmatory. Consequently, the question that needs to be answered is what number of studied subjects had abnormal cognitive and neurodevelopmental outcomes using standardized IQ testing. Secondly, ASQ score of studied subjects had not been compared with healthy control children. Thirdly, we did not consider mean time of normalization of T4 and TSH and parental socioeconomic status. Further studies in this regard are mandatory.

Finally, ASQ score of the relatively small sample size limits the study’s statistical power. These limitations require further investigation.

**In conclusion,** ASQ score was subnormal in 41% of treated patients with congenital hypothyroidism. Early diagnosis, early initiation of treatment, and initial L-thyroxin dose are important to optimal neurodevelopmental. The initial levothyroxine dose should be higher than 12 μg/kg/d. Confirmation and start of treatment of CH should be within the recommended time of 2 wk. However, the developmental status of studied subjects was measured using the ASQ screening methods, further studies should assess intelligence quotient (IQ) using standardized IQ testing.
